# Influence of the season on vitamin D levels and regulatory T cells in patients with polymorphic light eruption†
†Electronic supplementary information (ESI) available. See DOI: 10.1039/c5pp00398a



**DOI:** 10.1039/c5pp00398a

**Published:** 2016-02-25

**Authors:** N. A. Schweintzger, A. Gruber-Wackernagel, N. Shirsath, F. Quehenberger, B. Obermayer-Pietsch, P. Wolf

**Affiliations:** a Research Unit for Photodermatology , Department of Dermatology , Medical University of Graz , Graz , Austria . Email: peter.wolf@medunigraz.at ; Fax: +43 316 385-12466 ; Tel: +43 316 385-12371; b Center for Medical Research , Medical University of Graz , Graz , Austria; c Institute for Medical Informatics , Statistics and Documentation , Medical University of Graz , Graz , Austria; d Division of Endocrinology and Metabolism , Department of Internal Medicine , Medical University of Graz , Graz , Austria

## Abstract

The exact mechanisms of photohardening in polymorphic light eruption (PLE) are still unknown, but medical photohardening was shown to increase regulatory T cell (Treg) numbers in the blood of PLE patients, similar to natural hardening. Furthermore, oral vitamin D supplementation increased peripheral Tregs in healthy individuals. We herein report on a *post hoc* analysis of 26 screened PLE patients of a clinical trial (ClinicalTrials.gov No. NCT01595893), in which the influence of the progressing season was investigated on baseline CD4+CD25+FoxP3+CD127– Treg numbers by flow cytometry and Treg suppressive function by co-culture assays with T effector cells as a secondary endpoint, together with 25-hydroxy vitamin D (25(OH)D) serum levels at the study's screening visit, taking place in the period from January to June. The mean 25(OH)D serum level of all patients was 33.2 ng ml^–1^. Ten of those patients (38.5%) were identified with low 25(OH)D levels (<30 ng ml^–1^). Significantly higher baseline 25(OH)D serum levels (plus 34.4%; *P* = 0.0182) as well as higher relative Treg percentages in CD4+ population (plus 62.8%; *P* = 0.0157) and in total lymphocyte population (plus 59.6%; *P* = 0.0372) and higher absolute Treg numbers (plus 100.2%; *P* = 0.0042) were observed in the late spring/early summer period (April to June) compared to the winter period (January to February). No significant relationship was observed when Treg numbers and function were correlated with 25(OH)D levels. These data indicate that in PLE patients Treg numbers and their suppressive function are independent of vitamin D serum levels and suggest that UV light and/or other seasonal factors may affect these cells *via* the non-vitamin D related pathway(s).

## Abstract 2

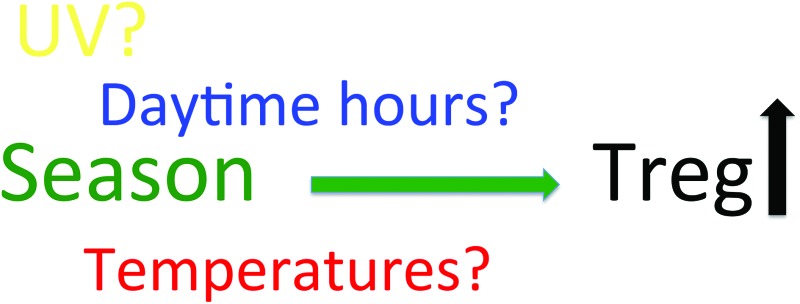
The levels of regulatory T cells (Tregs) are higher towards summer in patients with polymorphic light eruption, as a potential compensatory mechanism to suppress immune function. UV light and/or other seasonal factors may be responsible through the non-vitamin D related pathway(s).

## Introduction

Polymorphic light eruption (PLE) is a common seasonal photodermatosis that affects predominantly young women in the first decades of life.^1–6^ Itchy skin lesions of different morphologies are provoked by first intense sun exposure in spring or early summer and a so called “skin hardening effect” is usually observed during the summer season resulting in the development of tolerance even of higher sun dosages.^2,7,8^ Such a hardening can also be achieved in patients by different medical phototherapy modalities such as broadband UVB, narrowband 311 nm UVB or psoralen plus UVA (PUVA) photochemotherapy.^5,8,9^

The exact mechanisms leading to hardening remain unknown at present, but thickening of the stratum corneum, an increase in skin melanization and/or an immunological mechanism are thought to play a role.^10–12^ The current hypothesis of PLE pathogenesis is that UV exposure results in the formation of an immunogenic trigger (possibly a newly formed photoantigen) that initiates a cascade of events leading to the skin rash of PLE.^13^ It is thought that in normal healthy individuals such potential triggers upon UV irradiation are also formed, but the subsequent cascade is downregulated by simultaneous immunosuppression through UV and an immune response cannot be mounted.^5^ In contrast to PLE patients who show immunological abnormalities associated with dysregulated cytokine levels,^14,15^ Langerhans cell resistance^16,17^ and the deficiency of neutrophil infiltration^16,18,19^ upon UV radiation thought to be crucial in a resistance against UV-induced immunosuppression, as measured by suppression of the induction of contact hypersensitivity^20,21^ or induction of immunotolerance.^22^

CD4+CD25+FoxP3+ regulatory T cells (Tregs) are crucial in inducing immunotolerance^23,24^ and may be involved in the pathogenesis of PLE. Said so, decreased Treg infiltration was observed in UVA1-provoked skin lesions of PLE patients.^25^ Moreover, PLE patients showed an increase in Treg numbers together with a trend for improvement of the suppressive function after medical photohardening therapy.^26^ Serum levels of vitamin D correlated positively with the suppressive capacity of Tregs in multiple sclerosis patients, proposing vitamin D as an important mediator of T cell regulation *via* inhibition of Th1 and Th17 cells.^27–29^ Furthermore, Gruber-Wackernagel *et al.* ^30^ showed that pretreatment of the PLE-prone skin with a 1,25-dihydroxyvitamin D_3_ analogue (calcipotriol)-containing cream reduced the symptoms of the disease upon subsequent experimental photoprovocation in all patients tested in contrast to a vehicle-cream. Topical vitamin D analogues have known immunosuppressive properties^31,32^ and hereby may have acted in this study.^30^ Moreover, previous work, together with work from our laboratory has shown that patients with various photodermatoses including a part of tested PLE patients had low 25-hydroxy vitamin D (25(OH)D) levels.^33–35^ We therefore initiated a placebo-controlled clinical study to investigate the effect of oral vitamin D supplementation on PLE (ClinicalTrials.gov No. NCT01595893). However, this study had to be terminated prematurely since a majority (16/26 [61.5%]) of screened patients did not meet the main inclusion criterion which was a 25(OH)D serum level below 30 ng ml^–1^. Due to the low number of randomized patients, addressing the main original study aim, *i.e.* the effect of vitamin D supplementation on PLE susceptibility was not feasible. We herein report on the *post hoc* analysis of Treg numbers and function in all the screened PLE patients of the study in relation to 25(OH)D serum levels at the screening visit, taking place in the period from January to June. We observed that PLE patients displayed significantly higher 25(OH)D serum levels with progressing season, which was paralleled by absolute and relative Treg numbers. Higher Treg numbers were found to be independent of vitamin D.

## Results

### 25(OH)D serum levels

The mean 25(OH)D serum levels of all 26 patients were 33.2 ng ml^–1^. Sixteen of these patients (61.5%) displayed vitamin D serum levels above 30 ng ml^–1^ (mean 38.7 ng ml^–1^) and ten patients (38.5%) were identified with low 25(OH)D serum levels (<30 ng ml^–1^; mean 24.5 ng ml^–1^) that were subjected (according to the study protocol) to the study phase with photoprovocation and randomized administration of oral vitamin D or placebo (see ESI Table 1 and Table 2† for patient characteristics). The clinical trial was prematurely terminated after 26 patients had been screened, since it became evident that the majority of patients had 25(OH)D serum levels above 30 ng ml^–1^ and addressing the original study hypothesis (that oral vitamin D supplementation does protect against PLE) was neither reachable within a proper time frame nor appropriate and thus this analysis was not executed.

### Absolute and relative Treg numbers of PLE patients are higher towards summer

The analysis of this report therefore focuses on investigating a possible influence of season on baseline Treg numbers, Treg function and vitamin D serum levels in 26 PLE patients at the time point of first study visit (TP1), taking place in the period spanning from January to June, whereas the other time points were omitted from the present analysis (see the original study setup, ESI Table 1†). Grouping of patients at TP1 in two periods *i.e.* the winter period for those recruited from day 10 to 42 (January 10 to February 11) and the spring/early summer period for those recruited from day 108 to 176 (April 17 to June 24) allowed comparison of Treg numbers and function with respect to the season.

**Fig. 1:**
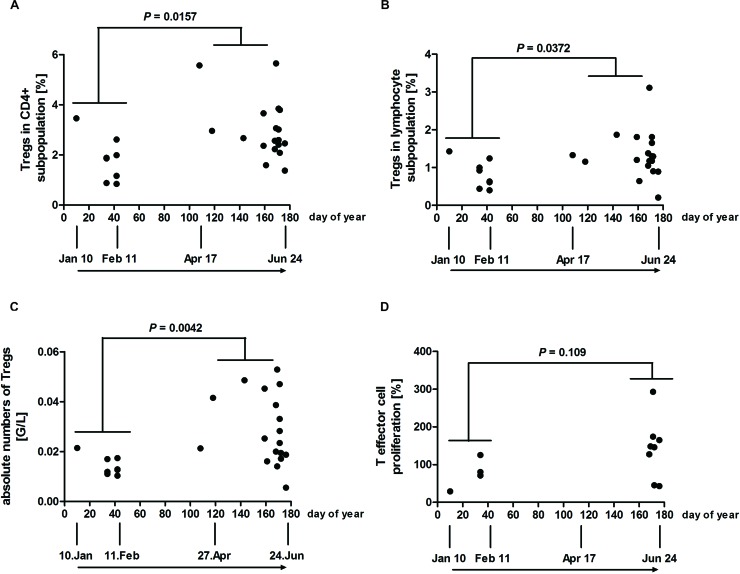
Tregs of the CD4+ subpopulation and absolute Tregs are higher in the summer period but show no significant increase in Treg function (*e.g.* less T effector cell proliferation). PBMCs of PLE patients were stained with antibodies for CD4, CD127, CD25 and FoxP3. Individual percentages of Tregs as a proportion of (A) CD4+ cells and (B) all lymphocytes are shown. (C) Individual absolute Treg numbers from PLE patients were determined by flow cytometry and calculated using lymphocyte counts by automated blood cell analysis. (D) Individual Treg suppression assays where data are shown as individual 1 : 1 ratios, normalised to T effector cell proliferation alone (0 : 1 ratio = 100%). 1 : 1 ratio represents the results of co-culture of equal numbers of T effector (CD4+CD25–CD127+) and Tregs (CD4+CD25+CD127–). All data are plotted against day of year. (A–C) *n* = 26 PLE patients; (D) *n* = 12 PLE patients. *P*-Values for comparison of the two periods (January 10 to February 11 *vs.* April 17 to June 24) were determined by using the Mann–Whitney test.

To study the influence of season (plotted as days of year in the graphs, spanning from January to June) on Tregs of PLE patients, their numbers were investigated by staining PBMCs for CD4, CD25, CD127 and FoxP3 as described.^26^ Flow cytometry analysis revealed significantly higher mean CD4+CD25+FoxP3+ Treg percentages (2.997 *vs.* 1.840%; plus 62.8%) towards summer in the CD4+ subpopulation (Fig. 1A), as well as in the total lymphocyte subpopulation (1.331 *vs.* 0.8338%; plus 59.6%) (Fig. 1B), by comparing values of the late spring/early summer period from day 108 to 176 (April 17 to June 24) with those of the winter period from day 10 to 42 (January 10 to February 11). Absolute mean Treg numbers were found to be significantly higher (0.02867 *vs.* 0.01432 G/L; plus 100.2%) towards summer (Fig. 1C). The possible influence of the season on the Treg suppressive function of PLE patients was investigated, measured *via* suppressive assays. T effector cell populations of eight patients did not show sufficient proliferation upon stimulation with CD3/CD28 antibodies (*i.e.* less than 10 000 corrected counts per million in the thymidine incorporation assay) and were omitted from this analysis (ESI Fig. 1†). In addition, six patients displayed a high variability in the duplicate measurements in the proliferation of Teff cells or the 1 : 1 co-culture (*i.e.* greater than 50% difference between duplicate measurements) and were also excluded from the present analysis. The Treg suppressive capacity of the remaining patients showed no statistically significant difference when the two periods were compared (Fig. 1D). Absolute numbers of neutrophil, eosinophil and basophil granulocytes, monocytes and total lymphocytes determined from PLE patients were also similar at the two periods (data not shown).

### Individual 25(OH)D serum levels of PLE patients are influenced by the season but do not correlate with Treg levels and function

**Fig. 2:**
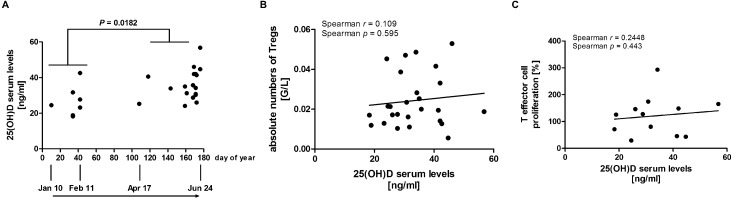
25(OH)D serum levels were significantly higher in late spring/early summer (April 17 to June 24) compared to the winter period (January 10 to February 11), but did not correlate with absolute Treg numbers and function in PLE patients. (A) Individual 25(OH)D serum levels were determined and are plotted *versus* day of year. (B) Absolute numbers of Tregs and (C) data from Treg suppression assays are plotted against their respective 25(OH)D serum levels. Data from (C) shown are individual 1 : 1 ratios, normalised to T effector cell proliferation alone (0 : 1 ratio = 100%). 1 : 1 ratio represents the results of co-culture of equal numbers of T effector (CD4+CD25–CD127+) and Tregs (CD4+CD25+CD127–). (A) and (B) *n* = 26 PLE patients; (C) *n* = 12 PLE patients. *P*-Values were determined using (A) Mann–Whitney test and (B) and (C) Spearman correlation.

**Fig. 3:**
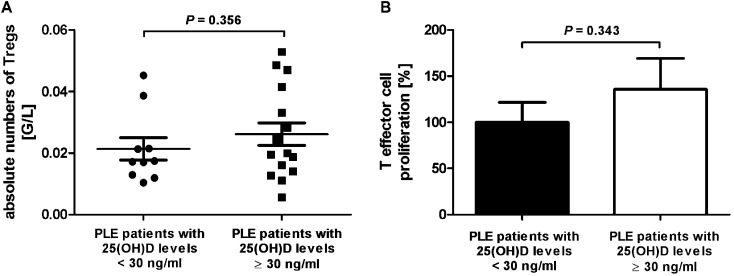
Treg levels and function are similar in PLE patients with 25(OH)D serum levels above or below 30 ng ml^–1^. (A) Absolute numbers of Tregs and (B) data of Treg suppression assays from patients with 25(OH)D serum levels below and at or above 30 ng ml^–1^ are shown. Data from (B) shown are individual 1 : 1 ratios, normalised to T effector cell proliferation alone (0 : 1 ratio = 100%). 1 : 1 ratio represents the results of co-culture of equal numbers of T effector (CD4+CD25–CD127+) and Tregs (CD4+CD25+CD127–). (A) *n* = 16 PLE patients with 25(OH)D serum levels at or above 30 ng ml^–1^ and 10 PLE patients with 25(OH)D serum levels below 30 ng ml^–1^; (B) *n* = 7 PLE patients with 25(OH)D serum levels at or above 30 ng ml^–1^ and 5 PLE patients with 25(OH)D serum levels below 30 ng ml^–1^. *P*-Values were determined by the Mann–Whitney test.

Significantly higher 25(OH)D serum levels were found towards June in PLE patients by comparison of the spring/early summer period with the winter period (mean value of 36.06 *vs.* 26.83 ng ml^–1^; plus 34.4%; Fig. 2A). No relationship between the absolute Treg numbers and 25(OH)D serum levels was observed when correlating these two parameters by Spearman testing (Fig. 2B). There was also no correlation between Treg suppressive function and 25(OH)D serum levels (Fig. 2C). Absolute Treg numbers of ten patients displaying low 25(OH)D levels (<30 ng ml^–1^) were compared with the numbers of patients displaying 25(OH)D levels at or above 30 ng ml^–1^. No significant difference of absolute Treg numbers (Fig. 3A) as well as relative Treg numbers (data not shown) was observed between these two groups of patients. The suppressive activities of Tregs from patients with low 25(OH)D levels and patients with 25(OH)D levels at or above 30 ng ml^–1^ did also not significantly differ (Fig. 3B).

## Discussion and conclusion

In previous studies we had investigated the effect of 311 nm UVB photohardening on 25(OH)D levels^33,34^ as well as Treg levels and function.^26^ In this study we analysed the potential effect of the season and found that PLE patients tested towards summer displayed higher relative and absolute Treg numbers as well as 25(OH)D serum levels compared to patients tested in the winter period. In addition we found that higher numbers of Tregs were independent of vitamin D. This is consistent with the results of the study by Smolders *et al.* ^27^ who found (despite a correlation with function) no correlation between 25(OH)D levels and the number of Tregs in multiple sclerosis patients, but contrasts the study by Bock *et al.* ^36^ where a high oral dose of vitamin D supplementation (140.000 IU per month) resulted in an increase in Treg numbers in apparently healthy individuals. The results of this study are substantiated by our previous report, where PLE patients exhibited an increase in Treg numbers during the progressing season, irrespective of whether they were subjected to medical photohardening or not.^26^ This contrasted the results in healthy subjects, in whom we did not observe a seasonal effect on Treg levels.^26^ Why PLE patients display dysregulated Treg numbers and/or an impaired Treg function is still unknown, but genetic factors^37–40^ or sex hormones^41,42^ may contribute to it and affect the susceptibility to UV-induced immunosuppression.

In this report, we found that 10 out of 26 (38.5%) PLE patients had an individual 25(OH)D serum value below 30 ng ml^–1^ in the period from January to June, a result consistent with a previous study from our laboratory, in which at maximum a third of PLE patients displayed such low serum levels.^34^ This contrasts the data obtained by Rhodes *et al.*,^35^ who found that patients with various photosensitive disorders appeared to have even lower 25(OH)D levels, ranging from 47% in summer to 73% of the patients in winter having levels below 20 ng ml^–1^. This difference may be explained by the different cohorts (PLE patients only *vs.* patients under various photosensitive conditions, including PLE, actinic prurigo, lupus erythematosus, photoaggravated eczema, psoriasis, and others), latitude and climate of Central Europe/Austria *vs.* UK, and/or methods of measuring 25(OH)D levels (chemiluminescence immunoassay *vs.* high-performance liquid chromatography) between the studies.^34,35^ The limitations of our study include the type of analysis applied (*post hoc*), the lack of monitoring or estimation of natural UV exposure, and the rate of failure of the Treg suppression assays.

Thus, at least in PLE patients any effect of UV radiation on Treg numbers and function may arise through non-vitamin D-dependent pathways. Alternatively, other (non-UV) factors may contribute to the affection of Tregs during the season. These factors may include other components of sunlight like visible light^43,44^ and infrared radiation,^45,46^ temperatures and the length of daytime hours.^47–50^

## Experimental

### Study design

This study was originally set up at the Photodermatology Unit, Medical University of Graz, Austria as a randomized, double-blinded placebo-controlled trail to assess the effect of oral vitamin D supplementation on the susceptibility to disease manifestation in patients with a history of PLE. The study was approved by the local Ethical Committee of the Medical University of Graz (application no. 24-220 ex 11/12) with the EudraCT-No. 2012-000300-15 (ClinicalTrials.gov No. NCT01595893). All patients provided written informed consent to participate and the study was conducted adherent to the Declaration of Helsinki principles. Key-eligibility criteria for the study enrolment were diagnosis of PLE which had to be confirmed by physician-guided patient's history, as previously described,^26^ phototesting procedures and/or histologic findings; age above 18 years; and good general health status. Exclusion criteria included intolerance to the vitamin D study medication (Oleovit D_3_™) or its solvent (coconut oil), presence or history of malignant skin tumours, dysplastic nevus syndrome, photosensitive diseases, sarcoidosis, renal insufficiency, topical vitamin D_3_ analogues within 3 months or oral vitamin D treatment within 6 months, antinuclear antibodies (anti-ds-DNA, anti-Ro/La), 25(OH)D levels (25(OH)D ≥30 ng ml^–1^) at the screening visit, hypercalcaemia >2.65 nmol L^–1^ in serum, ongoing treatment with thiazide diuretics or glycosides, systemic treatment with steroids or other immunosuppressive drugs within 4 weeks and direct UV irradiation of skin sites to be tested within 8 weeks before the trial start.

### Study subjects and procedures

Twenty-eight PLE patients were pre-screened and twenty-six patients (20 females and 6 males; mean age 46 years, range 24–76) were subjected to definite screening and first study visit between January and June of 2012 to 2014, before showing any manifestation of the disease in the season of enrolment. This first study visit has been defined as TP1 (see ESI Table 1†) and is graphically displayed in the figures as days of the year. At this visit 25(OH)D serum levels were determined and baseline flow cytometry and immune function studies were performed.

Two prescreened patients were not enrolled in the study. One patient was diagnosed with lupus erythematosus and one patient withdrew from the trial before any study procedure was done. Their data are not included in the patient demographics and study analysis (see ESI Fig. 1† for the flow diagram of patients).

### Blood sample collection

Blood was collected for flow cytometry and Treg suppression assays using lithium-heparin tubes (Vacuette®, Greiner Bio-One, Kremsmünster, Austria) and for 25(OH)D serum level determination in serum tubes (Vacuette®, Greiner Bio-One, Kremsmünster, Austria).

### Quantitative 25-hydroxyvitamin D_3_ determination

25(OH)D serum levels were determined with the fully automated, quantitative, chemiluminescent immunoassays IDS-iSYS 25-Hydroxy Vitamin D and IDS-iSYS 25-Hydroxy Vitamin D^S^ with a IDS-iSYS 25OHD Control Set on the IDS-iSYS Multi-Discipline Automated Analyzer (IDS plc, Boldon, UK) at the Department of Internal Medicine, Division of Endocrinology and Metabolism, Medical University of Graz, Austria. Though no common agreement on the optimal concentrations of 25(OH)D serum levels in healthy individuals exists, we placed the threshold at 30 ng ml^–1^, following the recommendation of the US National Osteoporosis Foundation setting a level of >30 ng ml^–1^ to be protective for bone health.^51^ This goes in line with the consideration of the US National Kidney Foundation stating 25(OH)D levels <30 ng ml^–1^ as deficient^52^ and the finding that calcium absorption increased with 25(OH)D concentrations up to ∼30 ng ml^–1^ and plateaued above that level.^53^

### PBMC sorting and flow cytometry

For Treg suppression assays peripheral blood mononuclear cells (PBMCs) were isolated by density gradient centrifugation using Lymphoprep™ (StemCell Technology, Grenoble, France). The antibodies used for sorting and flow cytometry have been published previously.^26^ For flow cytometry of Tregs cells were pre-gated on CD4 positivity. Calculation of absolute Treg numbers was based on Tregs in the lymphocyte subpopulation and the absolute numbers of lymphocytes given as giga per liter (G per L) from the blood counts determined with the Sysmex XE-2100 (Sysmex Co., Kobe, Japan).

### Treg suppression assays

Treg suppression assays were performed, as described.^26,54^ In brief, PBMCs were isolated with Lymphoprep™ (StemCell Technology, Grenoble, France) and stimulated with 5 μg ml^–1^ purified, plate-bound, low endotoxin/sodium azide-free mouse anti-human CD3 (clone UCHT1; BD Pharmingen, San Diego, CA) and 2.5 μg ml^–1^ anti-human CD28 (Clone 28.2) (BioLegend, London, UK) for 96 h. Cell proliferation was visualized *via* tritiated thymidine incorporation (1 μCi per [^3^H]thymidine) (Amersham Biosciences, Piscataway, NJ), added for the final 16 h of the 96 h incubation with Wallac 1450 MicroBeta® TriLux (Perkin Elmer, Brunn am Gebirge, Austria). For analysis, the 1 : 1 ratio (*i.e.* that of the co-culture of the same number of T effector cells [CD4+CD25–CD127+] and Tregs [CD4+CD25+CD127–]) was compared with the proliferative rate of stimulated T effector cells alone (normalised and set to 100%) to determine the suppressive capacity of Tregs.

### Statistical analysis

Statistical analyses were performed using the Spearman correlation and Mann–Whitney U test as appropriate for the data with Prism 5.0 (GraphPad software Inc., SD, California) or R 3.1.2 (http://www.r-project.org). Statistical significance was set at *P* < 0.05.

## Abbreviations

PBMCs: Peripheral blood mononuclear cells
PLE: Polymorphic light eruption
Tregs: Regulatory T cells
UV: Ultraviolet
