# Supplementary material for: Influence of the season on vitamin D levels and regulatory T cells in patients with polymorphic light eruption
Source: Photochem Photobiol Sci. 2016 Feb 25;15(3):440–6. doi: 10.1039/c5pp00398a (PMC4841162; doi:10.1039/c5pp00398a)
Supplement: Supplementary file 1 [file PP-015-C5PP00398A-s001.pdf]

Electronic Supplementary Material (ESI) for Photochemical & Photobiological Sciences.
This journal is © The Royal Society of Chemistry and Owner Societies 2016

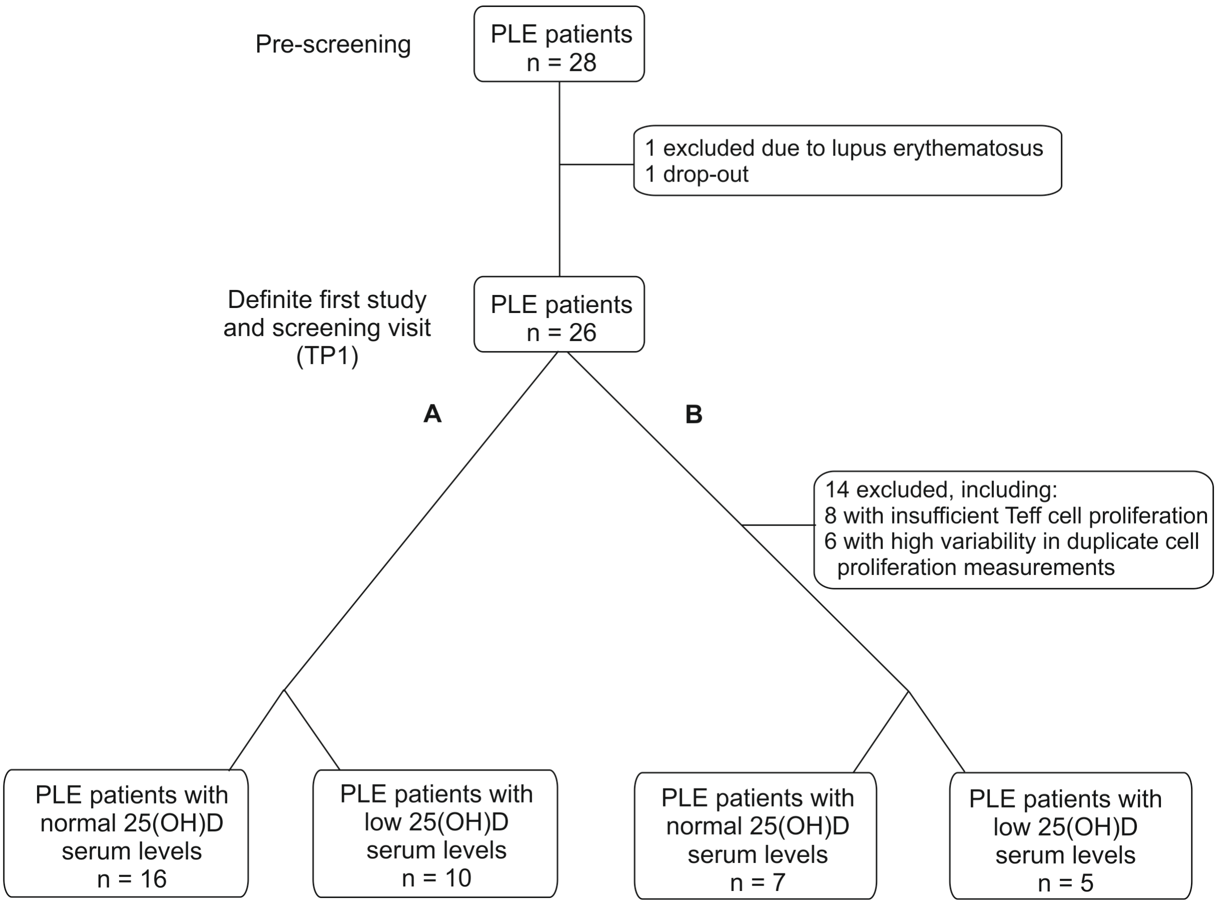

Supplementary Figure 1. Flow diagram showing numbers of PLE patients investigated at TP1 by (A) flow cytometry and (B) Treg suppression assays.
