# Supplementary material for: Influence of the season on vitamin D levels and regulatory T cells in patients with polymorphic light eruption
Source: Photochem Photobiol Sci. 2016 Feb 25;15(3):440–6. doi: 10.1039/c5pp00398a (PMC4841162; doi:10.1039/c5pp00398a)
Supplement: Supplementary file 2 [file PP-015-C5PP00398A-s002.pdf]

Electronic Supplementary Material (ESI) for Photochemical & Photobiological Sciences.
This journal is © The Royal Society of Chemistry and Owner Societies 2016

Supplementary Table 1. Time table of the original study

**Study day (time point)**	**Actions**
1 (TP1)	Blood sample collection for 25(OH)D determination, 1st MED testing
2 - 5	1st Photoprovocation testing
8	1st Photoprovocation reading, 1st administration of vitamin D or placebo
22 (TP2)	Blood sample collection, 2nd administration of vitamin D or placebo
36 (TP3)	Blood sample collection (TP3), 2nd MED testing, start of 2nd photoprovocation
43	2nd Photoprovocation reading, handing out of questionnaire

Supplementary Table 2. Characteristics of PLE patients.

Characteristics	Patients with normal 25(OH)D serum levels	Patients with low 25(OH)D serum levels
**Gender**	f: 12; m: 4	f: 8; m: 2
**Age in years (range)**	45.2 (24-68)	47.2 (25-76)
**Skin phototype*, n**	I: 1; II: 8; III: 6; IV: 1	I: 1; II: 2; III: 7
**Mean disease duration in years (range)**	21.2 (1-42)	14.6 (1-29)
**Establishment of diagnosis**	**PGPH: 16; CE: 7; P: 4; H: 1	PGPH: 10; CE: 5; P: 3; H: 3
**Results of photoprovocation (years before study start and at study start)**	*** UVA+/UVB+: 1; UVA-/UVB+:1; UVA-/UVB-: 2; nd: 12	UVA+/UVB+: 1; UVA-/UVB+: 1; SSR+: 1; nd: 7
**Pathology of PLE lesions**	**** mp: 2; pap: 6; pv: 7; v: 1	mp: 4; pap: 3; pv: 2; v: 1
**Predilection body site**	face: 2; V-neck: 16; arm: 10; forearm: 8; trunk: 10; thigh: 6; leg: 1; foot: 1	face: 0; V-neck: 9; arm: 4; forearm: 5; trunk: 0; thigh: 2; leg: 0; foot:2
**25(OH)D serum levels at TP1 (mean with range)**	38.7 (30.4-56.8)	24.5 (18.3-28.8)

* According to Fitzpatrick classification; ** PGPH = Physician-guided patient history, as described in Patients and Methods; CE = Clinical examination at presentation of the disease; P = Photoprovocation; H = Histology; *** SSR = solar simulated radiation; +, positive or -, negative for the different wavebands as indicated with laboratory photoprovocation with near-erythematogenic daily exposure over up to 4 days; nd = not done; **** mp = maculopapular; pap = papular; pv = papulovesicular; v = vesicular; abd. = abdomen
